# Developing a Virtual Reality Application for Social and Emotional Wellbeing and Cultural Determinants of Health Support With an Aboriginal Community of Sydney, New South Wales, Australia: Protocol for an Acceptability and Feasibility Study

**DOI:** 10.2196/88001

**Published:** 2026-06-15

**Authors:** Jasper Garay, Shane Phillips, Joseph Purdam, Jake Duczynski, Melissa Azizi, Michelle Dickson, Marcus Carter, Luke Hespanhol, Joel Negin, Julie Mooney-Somers

**Affiliations:** 1 School of Public Health, Faculty of Medicine and Health, The University of Sydney, Sydney, New South Wales, 2006, Australia, 61 2 9351 6645; 2 Tribal Warrior Corporation, Sydney, New South Wales, Australia; 3 Phoria, Melbourne, Victoria, Australia; 4 Studio Gilay, Sydney, New South Wales, Australia; 5 Sydney School of Architecture, Design and Planning, The University of Sydney, Sydney, New South Wales, Australia

**Keywords:** Aboriginal and Torres Strait Islander, social and emotional wellbeing, cultural determinants of health, mental health, virtual reality, public health, digital health, Indigenist, participatory action research, qualitative health research

## Abstract

**Background:**

As the first peoples of Australia, Aboriginal and Torres Strait Islander peoples have continuing cultures that are essential to wellbeing. Complex sociocultural, health, and wellbeing inequities stemming from colonization, settler-colonialism, and mental health system challenges have led to high rates of negative mental health and wellbeing for Aboriginal and Torres Strait Islander peoples. Improving Aboriginal and Torres Strait Islander mental health and wellbeing outcomes is a national public health priority. Social and emotional wellbeing (SEWB) and the cultural determinants of health (CDH) provide evidence-based approaches for providing culturally centered wellbeing support. There is a need to increase the availability, accessibility, and effectiveness of culturally relevant, holistic, and strengths-based wellbeing supports. It is essential that Aboriginal communities have self-determined opportunities to develop and implement culturally centered wellbeing supports informed by SEWB and the CDH. Aboriginal digital health and wellbeing support research is an emerging field offering potential to help improve wellbeing outcomes. This study aims to explore how virtual reality (VR) could be used to provide SEWB and CDH support for Aboriginal and Torres Strait Islander peoples.

**Objective:**

This study protocol outlines a 3-phase mixed-methods approach that will inform the co-design and codevelopment of a VR application that aims to provide SEWB and CDH support. In partnership with Tribal Warrior, an Aboriginal Community Controlled Organization, Studio Gilay, an Aboriginal-led animation and storytelling studio, and Phoria, an Australian immersive storytelling technology company, this study will assess cultural relevance, acceptability, and feasibility of the VR application.

**Methods:**

Using Indigenist and Participatory Action Research methodologies, purposive sampling will be used to recruit 35 Tribal Warrior staff and Aboriginal community members to participate in each phase of research. Qualitative data collection will occur in each phase through yarning circles. Reflexive thematic analysis will guide qualitative analysis. Phase 3 will involve a quantitative survey, generating cultural relevance, acceptability, and feasibility evidence. Descriptive statistics analysis will be used to report results.

**Results:**

As of April 2026, data collection and analysis for phases 1 and 2 are complete. This study will culminate in the development and assessment of a co-designed and codeveloped VR application that aims to provide SEWB and CDH support for Aboriginal peoples. Findings from each phase will be published in academic papers and nonacademic outputs. The VR application will be implemented by Tribal Warrior into existing community programs and supports.

**Conclusions:**

Findings from this study have potential implications for improving availability and accessibility to culturally centered wellbeing supports for Aboriginal and Torres Strait Islander peoples. Assessing the cultural relevance, acceptability, and feasibility of using VR technology to provide culturally centered wellbeing support will contribute novel evidence to the fields of public health, digital health, and design-based research.

## Introduction

### Background

**Figure 1.**
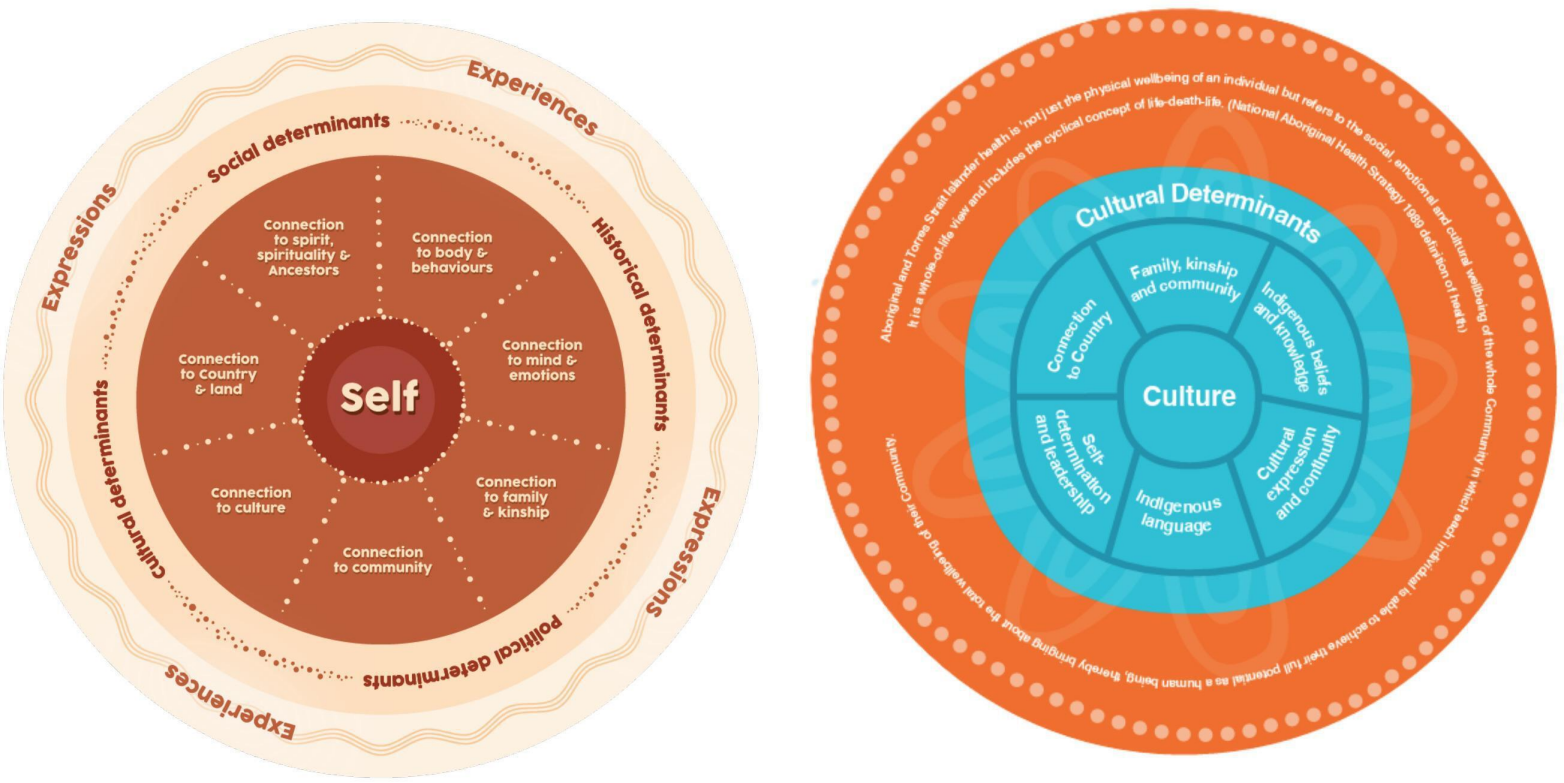
Social and emotional wellbeing and the cultural determinants of health. SEWB diagram adapted from Gee et al (2024) [16]. SEWB: social and emotional wellbeing.

As the first peoples of Australia, Aboriginal and Torres Strait Islander peoples (herein, respectfully referred to as Aboriginal peoples) have continuing cultures that are essential to wellbeing [1]. Social and emotional wellbeing (SEWB) and the cultural determinants of health (CDH) are models that conceptualize how culture supports Aboriginal health and wellbeing [2,3]. Comprised of cultural knowledges, practices, beliefs, and values, SEWB and the CDH provide evidence-based approaches for providing wellbeing support that is culturally centered [4,5]. Evidence suggests that culturally safe, holistic, and strengths-based approaches are essential factors of culturally centered wellbeing supports [6-9]. For Aboriginal peoples, supports targeting SEWB and CDH have been recognized as effective for improving wellbeing outcomes [10-13]. Improving the availability, accessibility, and effectiveness of culturally centered wellbeing supports for Aboriginal peoples is a longstanding national public health priority [5-7,10-14]. A visual overview of SEWB [15] and the CDH [9] is provided, as Figure 1 shows:

Culturally centered wellbeing supports help to counter the impacts of colonization and settler-colonialism [17-19]. It is well known that these factors are the underlying contributors toward unjust Aboriginal health, wellbeing, and social determinant disparities [20,21]. Government efforts to counter colonial harms have failed to address the racial oppression, cultural disconnection, and dispossession experienced intergenerationally by Aboriginal peoples [22-28]. In turn, Aboriginal communities have not been adequately afforded opportunities to provide culturally centered wellbeing supports that help to mitigate these challenges [29-32]. This has led to widespread experiences of complex mental health and wellbeing challenges for Aboriginal peoples. As of 2022‐2023, a total of 30% of the adult Aboriginal population experienced high or very high rates of psychological distress [33]. The largest contributors to the total burden of disease were mental and substance use disorders (23%), with anxiety and depressive disorders being the leading causes behind this outcome. Aboriginal males and females are respectively 2.9 and 2.6 times more likely than non-Indigenous peoples to have a cause of death by suicide [34]. For Aboriginal youth aged 10‐24, suicide and self-inflicted injuries made up the highest contribution toward the total burden of disease [35]. To improve Aboriginal wellbeing outcomes, mental health system reforms are required [36-39].

Culturally centered wellbeing supports for Aboriginal peoples are not adequately available in the Australian mental health system [10,11]. Despite a greater need for targeted wellbeing support, evidence has demonstrated that Aboriginal peoples are less likely to seek help when necessary [40-44]. Additionally, Aboriginal peoples experience subjective sociocultural challenges that impede engagement with available supports. Racial discrimination, lack of cultural safety, predominantly Western biomedical approaches to care, and an insufficient Aboriginal wellbeing workforce are key challenges [45,46]. When receiving support that is not culturally centered, feelings of shame and mistrust in non-Indigenous services often lead to disengagement with the mental health system [47-49]. Despite widespread awareness of these gaps, mental health system reforms have not led to sufficient improvements in Aboriginal mental health and wellbeing outcomes [50,51]. Increased investment in the co-design, implementation, and evaluation of culturally centered wellbeing supports is essential.

### Digital Health Applications and Aboriginal Wellbeing Support

Growing evidence demonstrates how digital health applications can offer benefits for Aboriginal wellbeing support [52-57]. Aboriginal digital health and wellbeing support studies have encompassed a wide range of technologies and definitions; electronic health, digital mental health services, telehealth, mobile health applications, and mobile diagnostic tools [52,58]. Engagement with diverse communities, technologies, research design, and wellbeing challenges is reported in the literature [59-61]. Aboriginal digital health and wellbeing support research, conducted in partnership with Aboriginal Community Controlled Organizations (ACCOs) and communities, has targeted psychological distress [60], suicide [62-65], anxiety and depression [66,67], and alcohol use [68]. Aboriginal self-determination and governance, genuine co-design, culturally relevant content, and strengths-based approaches are emphasized as essential factors of effective Aboriginal digital health and wellbeing support [52,58]. Fundamentally, digital health applications offer novel opportunities to provide culturally centered wellbeing support that is not currently available for Aboriginal peoples.

Digital health applications have been recognized as useful for addressing mental health system gaps and challenges impeding Aboriginal peoples seeking wellbeing support [55]. Structurally, digital health applications can improve accessibility to enhanced options of remote health care; supports that do not exist in communities can be provided, with reduced costs for patients [57,63]. Systemic challenges, such as racial discrimination, lack of cultural safety, and an inadequate Aboriginal wellbeing workforce, can also be countered [53,56,57,69]. In countering these challenges, digital health approaches can allow practitioners to provide remote support, informed by cultural safety, and holistic, strengths-based approaches [70]. Importantly, digital health applications can enhance access to culturally centered wellbeing supports, while Aboriginal peoples remain in Country and in community [52].

At present, gaps in knowledge exist within Aboriginal digital health and wellbeing support research [54]. There is a recognized need to better understand the cultural relevance, acceptability, feasibility, and effectiveness of digital health applications aiming to support Aboriginal wellbeing [56]. Addressing these gaps, the eHealth Research Collaboration for Aboriginal and Torres Strait Islander Health (eHRCATSIH) has conducted foundational research [53]. Established in 2019, the central aim of eHRCATSIH is to develop the first Aboriginal evidence-based best practice framework for culturally safe electronic health. Underpinning this work are review papers that have assessed the current state of Aboriginal digital health and wellbeing support research [52,54].

In a narrative review, important characteristics of electronic health interventions for Aboriginal peoples were assessed [52]. Of the 39 included studies, mobile health, telehealth, and mobile diagnostic tools were used to provide wellbeing support. Authentic co-design, Aboriginal governance, and partnerships were reported as foundational qualities of Aboriginal electronic health research and practice [52]. These qualities are recognized as essential for Aboriginal electronic health interventions to be culturally safe, sustainable, and impactful [52]. The necessity of prioritizing the development of research partnerships with ACCOs, using Indigenist and Participatory Action Research (PAR) approaches, is emphasized by eHRCATSIH [52].

Another systematic review examined the effectiveness, facilitators, and barriers of studies involving digital health mental services (DHMSs) for Aboriginal peoples [58]. Findings highlighted variations in the effectiveness of DHMS across mental health outcomes and interventions [58]. DHMSs were found to be effective supports for specific wellbeing support purposes: assessment of conditions, monitoring of status, and providing education [58]. While findings demonstrated DHMSs could improve general mental health support, limited evidence identified effective support for more severe conditions [58]. Key facilitators of effective DHMSs included Aboriginal community leadership and governance, culturally relevant design, and clinician-supported tools [58]. Barriers included digital exclusion, low literacy, and privacy issues [58].

Two Aboriginal digital health and wellbeing support studies exemplify this approach: iBobbly and Stay Strong. Both studies contribute valuable evidence reinforcing the importance of exploring, understanding, and assessing how digital health applications could support improved Aboriginal wellbeing outcomes. In partnership with Aboriginal community members in Western Australia, iBobbly was developed as a self-help mobile application based on acceptance-based therapies. iBobbly provided targeted support for Aboriginal youth wellbeing, addressing suicidal ideation, depression, psychological distress, and impulsivity. From a 2-arm randomized controlled trial involving 61 participants, a significant reduction in depression symptoms and psychological distress in the target population was reported [63]. As a co-designed tablet and mobile application, Stay Strong provides culturally adapted cognitive behavioral therapy (CBT), mindfulness-based activities, and trauma-informed wellbeing support for Aboriginal peoples. As a holistic wellbeing intervention, the Stay Strong app involves a gamified approach to support mental health literacy, emotional regulation, help-seeking, and goal setting. Stay Strong has been adapted to provide wellbeing support for specific subpopulations, including Aboriginal youth [62], people with kidney failure [61], and people in prisons [60]. In a nonrandomized prepost mixed methods study involving 30 Aboriginal young people, feasibility, acceptability, and use of the AIMhi youth Stay Strong app were assessed [67]. Findings showed statistically and clinically significant improvements in wellbeing measures for psychological distress and depression [67]. Participants reported that Stay Strong had good usability, was culturally relevant, and useful for wellbeing support. Although a diversity of digital health applications for wellbeing support have been explored in this context, evidence on the use of virtual reality (VR) to support Aboriginal wellbeing is minimal.

### VR and Indigenous Wellbeing Support

Research on the use of VR to support wellbeing has been conducted over many decades. Common themes of this research include mental health support [71-74], empathy and communication skills development [75-77], pain management [78-80], physiotherapy and rehabilitation [81-83], and surgical training [84-86]. VR offers specific affordances that can be leveraged to provide heightened psychological and physiological experiences [87]. Key affordances include immersion, presence, simulation, interactivity, and embodiment [88,89]. VR research has demonstrated that when meaningful sociocultural contexts are explored, and VR affordances are leveraged effectively, potential exists to influence emotions, perspectives, and wellbeing [90]. Yet for Aboriginal, First Nations, and Indigenous peoples (herein, respectfully referred to collectively as Indigenous peoples), the use of VR to support subjective sociocultural and psycho-social wellbeing has been largely overlooked.

Despite this, Indigenous peoples worldwide have been involved in VR uptake and development [91]. In 2021, analysis from FourthVR, an online Indigenous VR database, reported that 43 Indigenous VR works were available in the database [91]. From a paper involving analysis of 3 Indigenous VR works, recurring design and experiential themes were identified [91]. Embodying connections of the past-present-future, demonstrating interconnectivity of all living things, native languages in virtual worlds, and Indigenous futurism and activism were identified as recurring themes. These findings correlate with global conceptualizations of Indigenous wellbeing and align with evidence-based approaches to providing Indigenous wellbeing support [92-95]. Although some studies have begun to explore specific Indigenous VR wellbeing support use cases [96-99], many gaps within the evidence exist. Understanding how VR experiences and affordances might offer subjective wellbeing benefits for Indigenous peoples, and the clinical acceptability, feasibility, and effectiveness of VR wellbeing supports, are major gaps. At present, studies have not reported on genuine improvements in mental health and wellbeing outcomes through the use of VR.

However, 4 notable studies have begun to explore the potential of using VR to provide wellbeing support for Indigenous peoples. In 2024, the Transforming Trauma project received significant Australian federal government funding to develop Indigenous-led VR wellbeing support tools. In the coming years, this research will aim to provide trauma support through VR for Aboriginal peoples in regional and remote communities [100]. In partnership with ACCOs and wellbeing service providers, trauma support will be provided to young mothers and young men transitioning from the justice system. Future research will evaluate cultural, psycho-social, and health impacts from engagement with the VR applications. The Digital Songlines project approached VR as a medium for cultural heritage preservation and education [101]. Collaboration with Aboriginal communities involved collecting, designing, and sharing Aboriginal cultural heritage knowledge in VR. Research evidence reported on the development of community engagement protocols, culturally grounded methodologies, and development toolkits for VR [101]. In 2023, Digital Songlines partnered with Menzies School of Health Research and local mental health services to facilitate a pilot program, supporting Aboriginal youth wellbeing in the Northern Territory, Australia [102]. For Stolen Generation Survivors and families, VR was approached as a digital health tool conducive to healing from colonial traumas [98,99]. The Carrolup-Marribank mission site was reconstructed in VR, serving as a living digital memorial where support could be provided for cultural disconnection, loss of identity, and community displacement. Stolen Generation Survivors and Aboriginal communities co-designed the VR application, using lived experiences and cultural knowledges. Findings reported that the VR application was supportive as an emotive and healing mechanism for truth-telling and reconciliation [99]. In Quebec, Canada, research with Inuit communities of Nunavik led to the development of a co-designed culturally adapted VR-CBT intervention [96,97]. The research addressed 3 challenges limiting psychotherapy access for Inuit: geographic location and limited access to high-quality care, lack of cultural safety, and lack of Inuk therapists. Outputs from participatory co-design included a culturally adapted CBT manual and 2 complementary VR-CBT Inuit therapy environments [96,97]. Further research is underway through a proof-of-concept 2-arm randomized controlled trial involving 40 Inuit [96]. Evidence from this study will include feasibility, self-rated mental wellbeing, and objective psychophysiological measures, and the identification of primary outcome measures.

Based on Aboriginal digital health and wellbeing support evidence, including the use of VR applications, there is a clear direction for future research. Understanding how VR could be used to support Indigenous wellbeing requires partnerships with Indigenous communities, emphasizing cultural governance and leadership. Participatory research approaches need to identify the wellbeing support needs of Indigenous communities, and how VR could address gaps in mental health systems. Through co-design and codevelopment processes, cultural factors conducive to positive Indigenous wellbeing can be understood, enabling adaptation into VR application content. Cultural content for VR applications should align with evidence-based Indigenous wellbeing supports, allowing meaningful experiences to be provided for users. Resulting from the availability of VR applications designed to support wellbeing, research on cultural relevance, acceptability, feasibility, and effectiveness can then occur.

### VR and Culturally Centered Wellbeing Support

**Figure 2.**
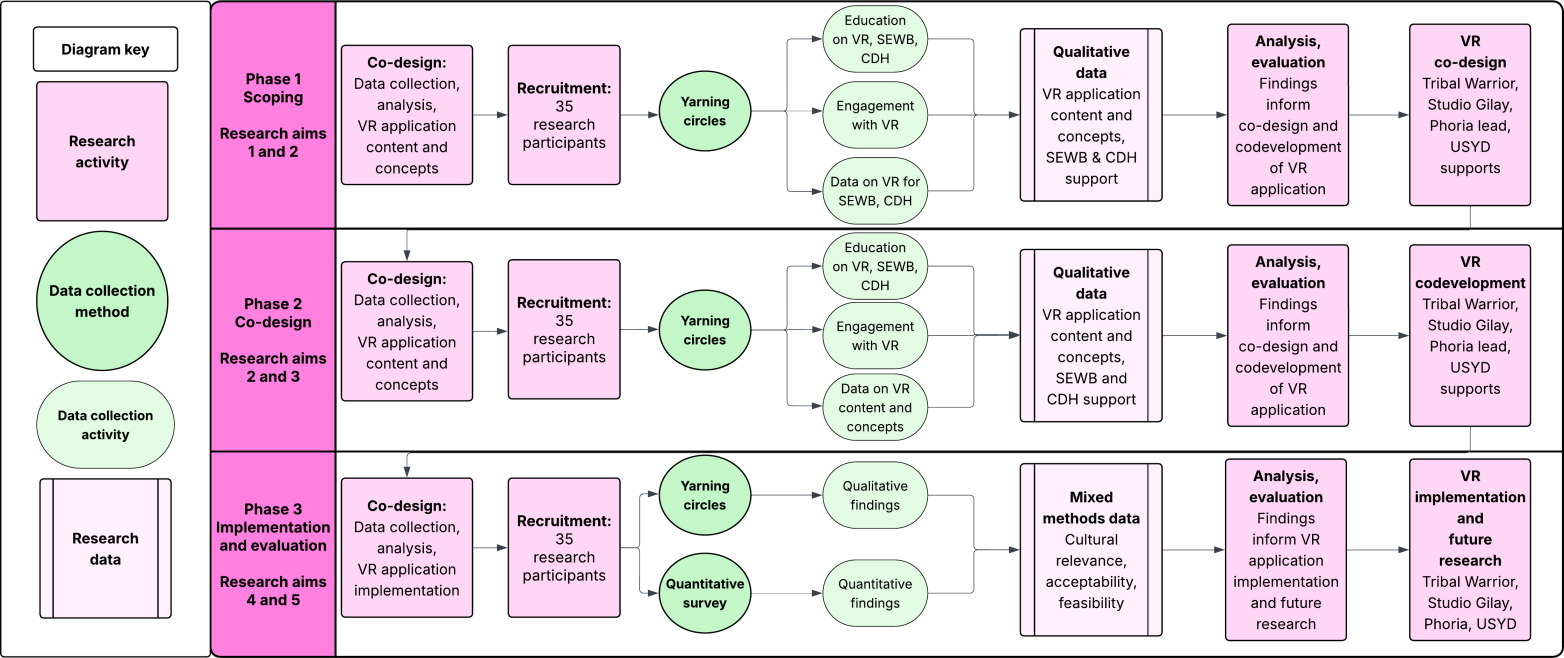
Research phases, aims, and methods. CDH: cultural determinants of health; SEWB: social and emotional wellbeing; USYD: University of Sydney; VR: virtual reality.

Our study will explore the potential of using VR to provide SEWB and CDH support for Aboriginal peoples. This protocol outlines a 3-phase mixed-methods indigenist and PAR cultural relevance, acceptability, and feasibility study. The central aim of this study is to co-design and codevelop a VR application that provides culturally centered wellbeing support, informed by SEWB and the CDH. Upon availability of the VR application, cultural relevance, acceptability, and feasibility will be assessed by participants. Cultural relevance will assess whether VR application experiences and content align with SEWB and CDH domains. Acceptability will assess whether the VR application experiences and content could be beneficial for providing SEWB and CDH support. Feasibility will assess how, where, and why implementation of the VR application could be effective as a digital health and wellbeing support application (Figure 2). The research aims and study design are provided below, as Table 1 shows.

**Table 1.** Research aims.

Aims	Description
1	Explore the perspectives of Aboriginal peoples on how VR^a^ could be used to provide SEWB^b^ and CDH^c^ support.
2	Use Aboriginal cultural knowledges and practices, sociocultural lived experiences of the Aboriginal community, and qualitative data to co-design and codevelop the VR application.
3	Assess the cultural relevance, acceptability, and feasibility of using the VR application to provide culturally centered SEWB and CDH support.
4	Support implementation of the VR application as a digital health application that facilitates SEWB and CDH cultural education and connection.
5	Engage in interdisciplinary and cross-industry knowledge translation and dissemination activities based on research findings.

a VR: virtual reality.

b SEWB: social and emotional wellbeing.

c CDH: cultural determinants of health.

### Research Partnership Positionality

Tribal Warrior Corporation (SP) is a nonprofit ACCO located in Gadigal Country, Eora Nation (Sydney, New South Wales, Australia). Tribal Warrior is directed by Aboriginal people to empower the community by supporting the health and wellbeing through connection to culture and family [103]. They provide targeted supports and programs across health, wellbeing, cultural education, justice systems, and tourism. Studio Gilay (JD and MA) is an Aboriginal-led animation storytelling studio, also based on Gadigal Country, Eora Nation. They prioritize respectful interpretation of voices, stories, and knowledges when consulting with diverse communities to enable the creation of meaningful digital content shared with broad audiences [104]. Phoria (JP) is an immersive technology studio based in Narrm, Wurundjeri Country, Kulin Nation (Melbourne, Victoria, Australia), specializing in the use of virtual and mixed reality technologies (VR, augmented reality, mixed reality, and extended reality) to generate social impact and achieve positive change [105]. Our research team and project at The University of Sydney (USYD) is led by Aboriginal Australian authors (JG and MD) and supported by non-Indigenous authors (JM-S and JN), who work in the Sydney School of Public Health and have expertise in public health and qualitative health research. Non-Indigenous authors (MC and LH) working in the School of Architecture, Design, and Planning at the USYD provide expertise in human-computer-interaction and design-based research. We position our research partnership as an interdisciplinary, cross-industry, innovative collaboration.

Tribal Warrior owns the full intellectual, cultural, and economic rights to the VR application and research data. Phoria and Studio Gilay do not gain financial profit from the VR application or research outputs. Both partner organizations will be provided with funding from the research grant and Tribal Warrior to complete co-design and codevelopment of the VR application . Funding was provided by Meta as an “unrestricted free gift.” This grant structure provides full autonomy for the research partnership to independently conduct research and develop the VR application without any involvement or expectations of Meta, Australia.

### Theoretical Approach: Using VR to Provide SEWB and CDH Support

This study starts from the position that VR could serve as a culturally centered wellbeing support mechanism. Findings from SEWB and CDH research demonstrate that cultural education and connection activities are essential factors for Aboriginal wellbeing support [6,106]. Arts-based programs, engaging with Elders, and empowering connections within the community have been recognized as effective modalities and approaches [107,108]. In theory, this evidence signifies that the experiential nature of VR could be useful for providing culturally centered wellbeing support for Aboriginal peoples. Co-design and codevelopment would enable the VR application to provide supportive wellbeing experiences informed by SEWB and CDH. Qualitative evidence, provided by Aboriginal peoples, could identify SEWB and CDH supports required in the community context, outlining effective modalities and approaches. Complementing this evidence, ACCOs could provide expertise and experiences of providing SEWB and CDH supports, further identifying those currently unavailable or ineffective. Together, cultural knowledges, practices, and contexts would inform VR application experiences, content, and use cases that allow the provision of relevant culturally centered wellbeing support.

As a mechanism of providing culturally centered wellbeing support, informed by SEWB and CDH, VR applications could counter gaps in mental health systems and ACCO supports. By addressing challenges and barriers of colonization and settler-colonialism, VR applications could serve as a novel approach to providing self-determined SEWB and CDH support. Therefore, the VR application would be adapted into existing ACCO services, programs, and supports as a new approach to providing SEWB and CDH support. Thus, this study aims to explore how VR applications could be used to provide SEWB and CDH support for Aboriginal peoples.

To be effective, VR applications providing SEWB and CDH support would need to leverage the affordances of VR [88,90]. This study theorizes that VR affordances, when leveraged within supportive wellbeing experiences targeting SEWB and CDH, could offer subjective benefits for Aboriginal peoples. Cultural contexts that no longer exist, or have been negatively impacted, could be adapted into immersive VR environments. Embodiment of avatars, or being positioned in specific viewpoints, could provide experiences that help Aboriginal peoples to experience presence, reconnecting with traditional contexts and cultural components precolonization. Specific simulations could be embedded, where Aboriginal peoples could benefit from interactivity with cultural practices and sociocultural histories. This could help to counter cultural disconnection by enhancing access to opportunities for cultural engagement and strengths-based perspective-taking using VR. By leveraging specific VR affordances to provide cultural experiences and content informed by SEWB and the CDH, the VR application could serve as a mechanism for providing culturally centered wellbeing support. This study will explore this theoretical approach, as outlined in the research aims.

### Research Design: Indigenist Methodologies

**Figure 3.**
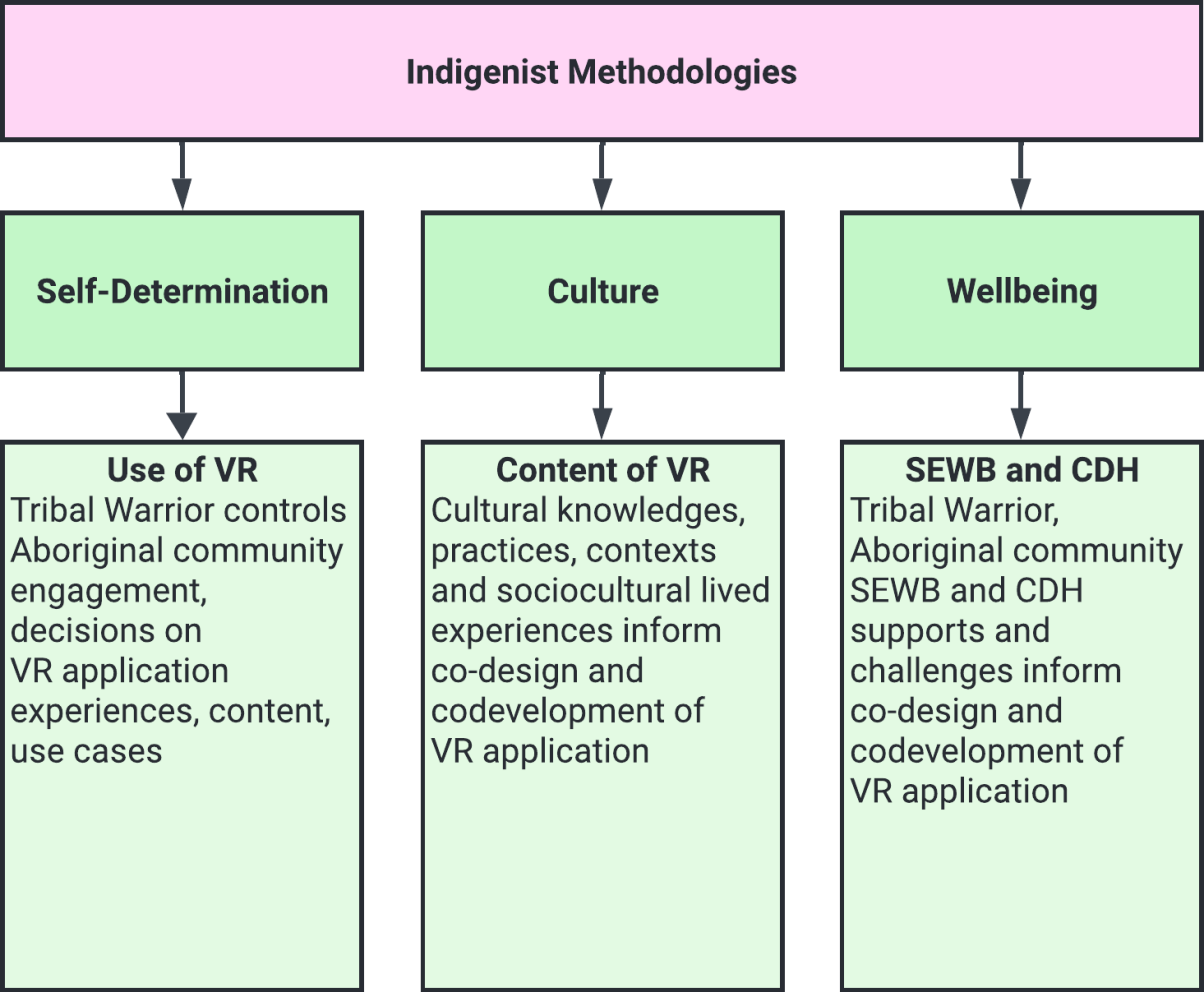
Research facilitation—participatory action research methodology. USYD: University of Sydney; VR: virtual reality.

Indigenist research methodologies offer beneficial processes for research design, engagement, and facilitation for Indigenous peoples and researchers [109]. In Australia, the concept of Indigenist research emerged as a collaborative effort between Indigenous researchers and communities. Promoting self-determination, decolonization, and liberation from colonial oppression, the interconnected principles of resistance, political integrity, and prioritizing Indigenous voices are foundational principles of Indigenist research [110]. Indigenist approaches emphasize support for Aboriginal worldviews, knowledge systems, and social norms taking precedence in research, helping to ensure Indigenous perspectives are not subordinated to Western research standards [111]. Internationally, Indigenist methodologies with similar interpretations and applications have been endorsed by numerous Indigenous and First Nations scholars [112-115]. Through Indigenist research, emphasis is placed on equitable involvement of all peoples. This enables Indigenous communities to meaningfully contribute toward decision-making, governance, and leadership across all research components [116-119]. Indigenist methodologies were chosen as the most suitable methodological approach to inform our research design. This respects and enacts self-determination for Tribal Warrior and Aboriginal community members within this study. Our Indigenist research design will ensure the VR application is co-designed and codeveloped from a culturally centered positionality. Qualitative findings and iterative co-design outcomes will inform VR application content codevelopment, prioritizing cultural knowledges, lived experiences, SEWB, and CDH support needs. Our use of Indigenist methodologies will ensure that the work of USYD researchers, Studio Gilay, and Phoria is responsive to self-determined decisions made by Tribal Warrior. This governance and leadership commitment will enable the partnership to align VR application co-design and codevelopment with how Tribal Warrior provides SEWB and CDH support for the local Aboriginal community. A visual overview of how Indigenist methodologies inform the research design is provided, as (Figure 3) shows:

### Research Facilitation: PAR Methodology

**Figure 4.**
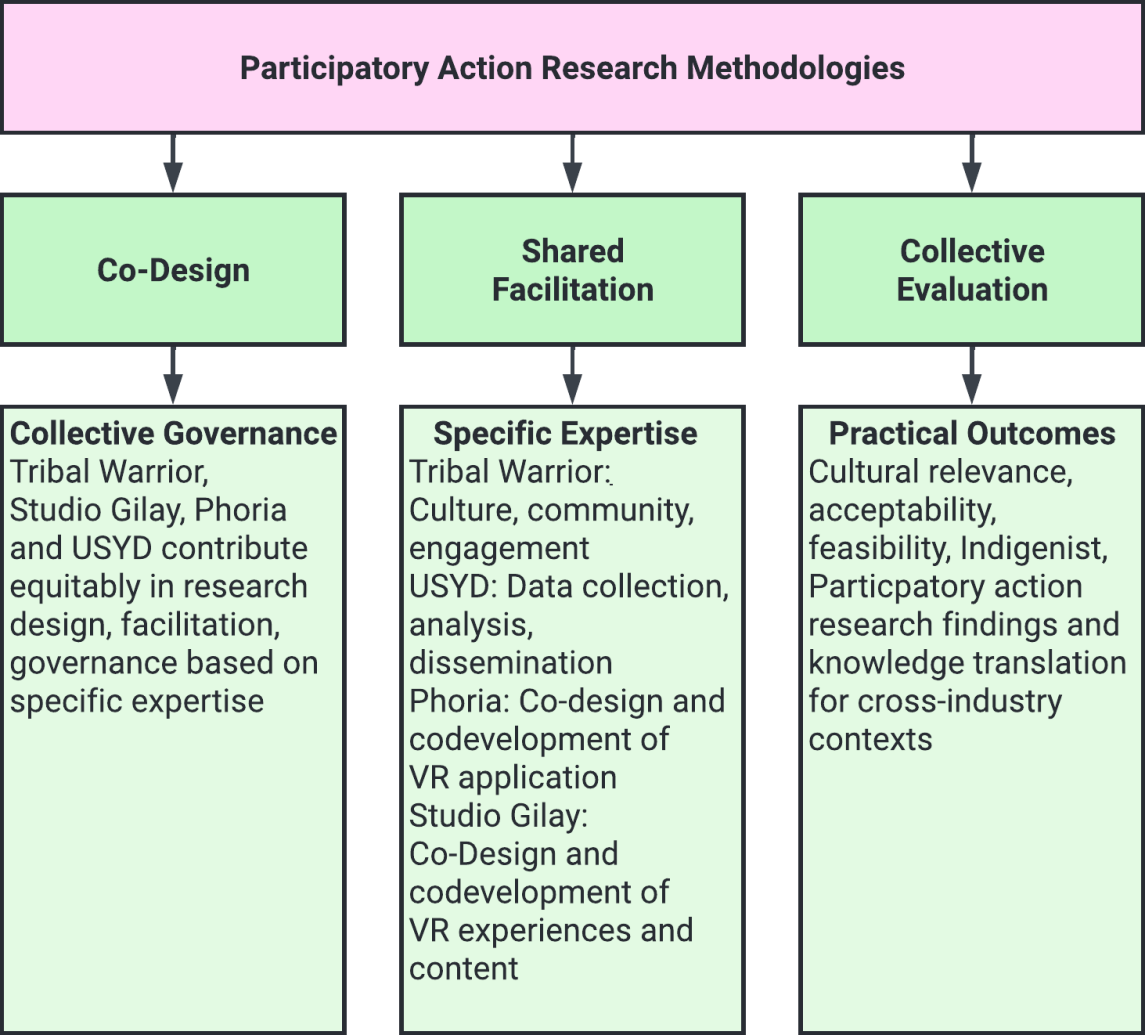
Research design–indigenist methodologies. CDH: cultural determinants of health; SEWB: social and emotional wellbeing; VR: virtual reality.

PAR methodologies emphasize the central role of research participants. PAR promotes that consistent and equitable participant involvement across all components of research is necessary for ethical, sustainable, and impactful research [120-122]. This requires the prioritization of forming practical partnership dynamics, having relational accountability, and consistently communicating progress or challenges with all involved in the research. PAR processes operate cyclically, encompassing reflection, planning, action, observation, and subsequent action. For participants, this means consistently providing leadership and governance, aiming to produce relevant and beneficial outcomes of importance to participant needs and contexts [123]. PAR expects that all stakeholder perspectives are valued equally across all components of research [124,125]. For research aiming to explore, comprehend, and define potential improvements with specific people and contexts, PAR is recognized as a suitable methodology. Furthermore, PAR has been identified as an effective method for conducting research with Aboriginal, Indigenous, and First Nations peoples. Several successful applications of PAR have been documented across various health and wellbeing projects involving Aboriginal, Indigenous, and First Nations peoples [126-138]. PAR methodologies will inform research facilitation of this study, ensuring Tribal Warrior staff and Aboriginal community members are involved across all components of research. For Tribal Warrior, our PAR approach will ensure all research planning, facilitation, co-design, and codevelopment of VR application content will occur based on constant communication, integration of feedback, and shared decision-making. The use of our PAR approach for research facilitation will uphold respect for the expertise of Tribal Warrior, an ACCO with longstanding expertise in supporting SEWB and CDH of the local Aboriginal community. Additionally, our PAR approach will assist the integration of SEWB and CDH support into the VR application, based on the lived experiences and perspectives of Aboriginal community members. A visual overview of how PAR methodologies inform the research facilitation is provided, as shown in (Figure 4):

## Methods

### Study Design

This study design includes a 3-phase mixed-methods co-design approach: phase 1 is regarding scoping; phase 2 is regarding co-design; and phase 3 is regarding implementation and evaluation. Participants will include 35 Tribal Warrior staff and/or Aboriginal community members. Qualitative yarning circles will generate data in each phase. Phase 1 thematic findings will guide iterative VR application co-design and codevelopment meetings before phase 2. Cultural knowledges, sociocultural lived experiences, SEWB, and CDH support the needs of the Aboriginal community and will inform the content of the VR application. Phase 2 will provide participants with opportunities to assess and give feedback on low-fidelity digital assets of the VR application. Thematic findings and further iterative co-design meetings will enable the redesign of content to be included in the fully developed VR application. Phase 3 will involve mixed-methods cultural relevance, acceptability, and feasibility assessment of the VR application.

### Co-Design and Codevelopment of the VR Application

**Figure 5.**
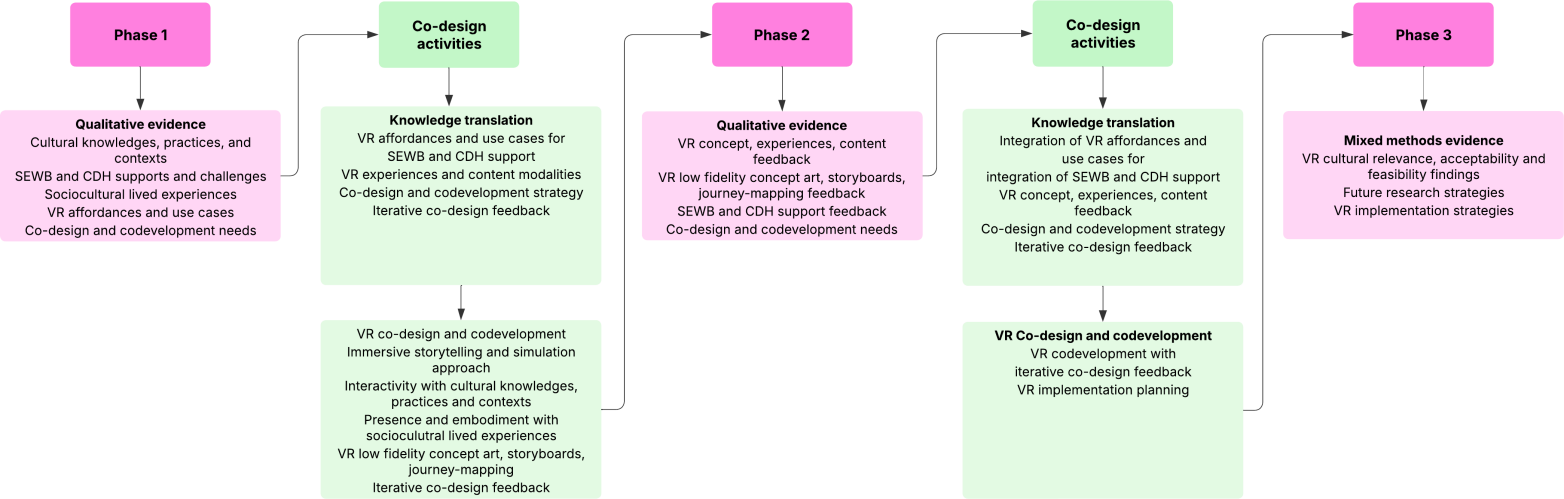
Virtual reality application co-design and codevelopment process. CDH: cultural determinants of health; SEWB: social and emotional wellbeing; VR: virtual reality.

Figure 5 shows how we codevelop the VR application. Our partnership will use an iterative co-design process. Phase 1 of qualitative findings will inform each co-design activity, ensuring participant evidence guides integration of cultural, SEWB, and CDH content. Findings from phase 1 will be reviewed during partnership knowledge translation meetings. Phoria will support Tribal Warrior to understand how certain VR affordances and modalities could be used to codevelop specific content conducive to providing SEWB and CDH support. Low-fidelity digital assets will then be codeveloped by Phoria, based on knowledge translation outcomes provided by Tribal Warrior. These will include concept art, storyboards, and journey-mapping outputs. Integration of feedback on content will occur until approval to proceed with VR application codevelopment is provided by Tribal Warrior. At this point, the VR application concept, content, SEWB, and CDH support experiences will be finalized. Phase 2 will generate feedback on these outputs, as well as exploring potential implementation and use case scenarios for the VR application. Repeating the above iterative co-design process, evidence will be integrated into the codevelopment of the VR application, led by Phoria and Studio Gilay. Regular partnership knowledge translation meetings will ensure codevelopment of content aligns with evidence from phases 1 and 2 and the direction provided by Tribal Warrior. Upon availability of the fully developed VR application, phase 3 will generate mixed-methods evidence of cultural relevance, acceptability, and feasibility. A visual overview of this theoretical approach is provided in the logic model, as Figure 5 shows :

### Data Collection: Mixed Methods

Qualitative data collection will occur through yarning circles in each phase. Yarning is a culturally specific qualitative research method often used when working with Aboriginal peoples [139]. As a semistructured and circuitous research method, yarning helps to facilitate supportive relational and communication processes between Aboriginal research participants and researchers [140]. Through yarning, Aboriginal research participants are supported within culturally safe and strengths-based data collection environments. Having these environments assists participants in sharing subjective cultural knowledge and lived experiences of relevance to the research [141]. Yarning involves 4 interdependent phases of relationality and communication (social yarning, research yarning, therapeutic yarning, and collaborative yarning), which concurrently support meaningful research engagements and data collection [142]. Due to our participants being Tribal Warrior staff or Aboriginal community members, the method of yarning circles was selected.

Aboriginal researchers (JG and MD) from the USYD will lead facilitation of yarning circles, as they have expertise with this research method. Non-Indigenous staff from Phoria (JP), responsible for leading co-design and codevelopment of the VR application, will be involved through observation during phase 1 and 2 data to ensure the VR technological, design, and implementation support needs of Tribal Warrior are integrated into iterative co-design activities. At least 1 Aboriginal researcher, 1 male, and 1 female, will be present during data collection, to uphold gendered and cultural representation. Yarning circles will be approximately 2 hours for each phase, taking place on campus at the USYD, at the office of Tribal Warrior, or at a location in the local Aboriginal community deemed suitable by Tribal Warrior. Audio recordings of each yarning circle will be transcribed using a professional and confidential academic service.

Quantitative data will be collected in phase 3 to generate evidence about the cultural relevance, acceptability, and feasibility of the VR application. Collecting quantitative data through the phase 3 survey provides an opportunity to capture perspectives of individual participants regarding cultural relevance, acceptability, and feasibility of the VR application. REDCap (Research Electronic Data Capture; Vanderbilt University) software will be used to design the survey, collect data, and perform analysis [143]. Participants will complete the survey on iPads (Apple Inc), laptops, or mobile phones. The results will be presented using a summary table in the phase 3 paper. The phase 3 survey questions and associations to cultural relevance, acceptability, and feasibility are provided below, as Table 2 shows:

**Table 2.** Phase 3 survey questions.

Questions	Characteristics
Question 1: what is your age? (years)	13‐24 25‐34 35‐44 45‐54 55‐64 65‐74 75 and older
Question 2 is regarding acceptability and feasibility: do you think the VR^a^ application could be used as an effective SEWB^b^ and CDH^c^ support for Aboriginal peoples?	Yes Unsure No
Question 3 is regarding cultural relevance: from the list below, which SEWB domains did the VR application explore?	Connection to country and land Connection to culture Connection to spirit, spirituality, and ancestors Connection body and behaviors Connection to mind and emotions Connection to family and kinship Connection to community
Question 4 is regarding cultural relevance: from the list below, which CDH domains did the VR application explore?	Family, kinship, and community Indigenous beliefs and knowledges Cultural expression and continuity Indigenous language Self-determination and leadership Connection to country
Question 5 is regarding acceptability and feasibility: from the list below, in which contexts do you think the VR application could be used to provide SEWB and CDH support for Aboriginal peoples?	Personal use at home Aboriginal community-controlled organizations Aboriginal medical services Mental health services Virtual therapy Hospitals/emergency departments Rehabilitation programs Youth justice or prisons Schools or universities Professional workplaces Cultural competency training Online social spaces Other: (free text entry)

a VR: virtual reality.

b SEWB: social and emotional wellbeing.

c CDH: cultural determinants of health.

### Data Analysis, Rigor, and Handling

Data analysis will be led by USYD researchers. The reflexive thematic analysis by Braun and Clarke [144] will guide qualitative data analysis. Inductive analysis will be used for each yarning circle transcript [145]. A total of 2 Aboriginal and 2 non-Indigenous researchers will undertake preliminary open-ended coding of 2 transcripts. Collaborative yarning meetings will review preliminary findings, assessing similarities and differences in coding outcomes from all 4 researchers [146]. Any disagreements or differences resulting from reflexive thematic analysis will be resolved during collaborative yarning meetings. Upon agreement on preliminary coding and analysis insights, broader themes will be established to guide further analysis. Further data analysis will then be conducted by researchers using NVivo software (version 15; Lumivero) [147]. The lead researcher (JG) will continue with reflexive thematic analysis for each transcript, based on the themes established in collaborative yarning meetings. At least 1 other researcher will be involved in the analysis of all transcripts. When analysis of each phase is complete, USYD researchers will engage in knowledge translation meetings with Tribal Warrior, Studio Gilay, and Phoria staff. As part of the iterative co-design process, findings will be used to support Tribal Warrior with co-designing and codevelopment of VR application content. Participants will be provided with 1-page plain English summaries of findings when findings are shared in academic papers.

In phase 3, descriptive statistics analysis will be used to summarize frequencies (n) and percentages (%) of participant responses to each survey question and response option [148]. Analysis and interpretation of survey data will provide cultural relevance, acceptability, and feasibility evidence. Cultural relevance will be assessed through questions 3 and 4, quantifying participant perspectives on which SEWB and CDH domains were engaged with through the VR application experience. Acceptability and feasibility will be assessed through questions 2 and 5, quantifying whether participants accept that the VR application could be used to provide SEWB and CDH support, and in which contexts this could feasibly occur.

### Recruitment and Sampling

Purposive sampling will be used, aiming to recruit the same 35 participants for each phase [149]. It may be necessary to recruit different Tribal Warrior staff and/or Aboriginal community member participants across phases, due to organizational changes and the unavailability of Aboriginal community members. However, all participants who become involved in this study will receive invitations to participate in future phases of data collection. Dropouts across phases will not negatively influence data collection, as involvement of different participants will increase the diversity of knowledge, perspectives, and lived experiences shared during data collection. Tribal Warrior will make self-determined decisions on which participants are recruited in each phase. All communication and engagement with potential participants will be managed by Tribal Warrior. This approach enacts self-determination and allows existing community connections and networks to be used effectively for recruitment. The sample size was selected intentionally, with Tribal Warrior confirming this approach would not be burdensome, enabling approximately one-third of all Tribal Warrior staff to be involved. Logistically, this sample size permits each yarning circle to involve 4 participants, using 4 VR headsets, in a timely yet meaningful manner. Further, this sample size aligns with previous Aboriginal wellbeing qualitative research conducted by the authors and across other Aboriginal wellbeing studies. Participant eligibility includes being a Tribal Warrior staff member and/or identifying as an Aboriginal community member. Diversity among participants will be prioritized, aiming to include representatives from each Tribal Warrior support program team and Aboriginal community members from differing cultural communities, lived experiences, ages, and genders. Ineligibility for our research includes being younger than 13 years of age [150], due to current health and safety advice on ethical use of VR hardware for young people and children. Upon confirmation of participants by Tribal Warrior, USYD researchers will provide plain English 1-page documents outlining each phase of research, how to become involved, and who to contact from the research team for further information. Upon confirmation, USYD researchers and Tribal Warrior staff will communicate details on expectations, timing, location, and phase-specific details. In each phase, participants will receive an Aus $200 (a currency exchange rate of Aus $1 = US $0.71 was applicable) visa-gift card for their time and contributions.

### Ethical Considerations

Our research has ethics approval from the Aboriginal Health and Medical Research Council of New South Wales, project number “2070/23.” From this, ethics approval was endorsed by the USYD Human Research Ethics Committee. Aboriginal Health and Medical Research Council principles and the Australian Institute of Aboriginal and Torres Strait Islander Studies code of ethics will be upheld during our research [151,152]. Data collected during this research will be stored safely and securely, as per the USYD Research Data Management Policy [153]. All Tribal Warrior staff and Aboriginal community members have the right to withdraw consent for any reason, up to the point of data analysis. All data used in academic publications and any output resulting from the research will be deidentified. Ethical facilitation of research will be ensured, prioritizing self-determination, cultural safety, and strengths-based approaches when engaging with Tribal Warrior. Owning all intellectual, economic, and cultural properties resulting from the research, Tribal Warrior will control all co-design, codevelopment, and implementation decisions for the VR application. It is the responsibility of each research partner to provide expertise toward these aspirations. Co-design and codevelopment progress will be reported consistently, enabling iterative communication and feedback outcomes to inform the VR application. Regular meetings with all research partners will include discussion and resolution of any potential ethical challenges or arising issues.

Our research team acknowledges that perceived conflicts of interest exist within the research partnership. Tribal Warrior’s ownership of all intellectual, economic, and cultural properties negates any potential motivations for Phoria and Studio Gilay to inflict bias during co-design and codevelopment. Specific Phoria staff have ethics approval to assist with co-designing and observing data collection. This ensures that the VR technological, design, and implementation support needs of Tribal Warrior can be understood and factored into iterative co-design activities. Phoria and Studio Gilay will not participate in data collection and analysis. Fundamentally, the research partnership has been designed to co-design and codevelop the VR application based on the self-determined guidance of Tribal Warrior as an ACCO.

## Results

### Overview

As of April 2026, data collection and analysis for phases 1 and 2 are complete. Tribal Warrior, Studio Gilay, and Phoria have used these findings to inform iterative co-design and codevelopment processes of the VR application. Phase 3 data collection is planned for June 2026. Upon availability of the VR application, academic papers will be submitted. Nonacademic outputs will be shared with Tribal Warrior and participants.

### Dissemination and Translation Plans

Knowledge translation and dissemination activities will center on the strategic implementation of the VR application by Tribal Warrior. Phase 3 findings will provide cultural relevance, acceptability, and feasibility evidence, informing knowledge translation meetings and implementation planning. Upon availability of the VR application, academic and nonacademic written outputs will be shared with interdisciplinary and cross-industry stakeholders. Academic papers from each phase of research will be published in peer-reviewed journals, with a focus on open-access papers to maximize accessibility. Community-focused dissemination outputs will include plain English language written documents and mixed-media outputs. These will focus on explaining the research, the intention of the VR application, user manuals, and implementation plans. Presentations at interdisciplinary conferences will engage broader research and industry community stakeholders. Conference presentation delivery will be led by Tribal Warrior staff and Aboriginal researchers. As a research partnership, we aspire to engage with future targeted research, building on the cultural relevance, acceptability, feasibility, and mixed-methods evidence generated through this study.

## Discussion

### Anticipated Findings

The results from this study will contribute novel evidence on the cultural relevance, acceptability, and feasibility of using VR to support Aboriginal wellbeing. To the best of our knowledge, this is the first Australian research engaging with an urban ACCO and Aboriginal community to explore the use of VR for Aboriginal wellbeing support. Cultural relevance, acceptability, feasibility, and wellbeing support findings will help to inform similar research in the future. Phase 1 findings could present implications for how VR applications are understood as a digital health wellbeing support application for Aboriginal peoples. This study will advance knowledge on how SEWB and CDH education, immersion, and connection can be facilitated through digital health application co-design and codevelopment. Phase 2 findings will contribute evidence on ethical Indigenist and PAR co-design and codevelopment processes when working with ACCOs and Aboriginal communities. Both strengths and challenges will be reported, enabling future research and partnerships to benefit from practical insights. Phase 3 findings will share cultural relevance, acceptability, and feasibility evidence, helping to understand how VR could be used to provide SEWB and CDH support for Aboriginal peoples. Future research will be conducted based on these collective results.

### Limitations

As this is an Indigenist and PAR study, data collection, analysis, and publication processes may need to be deprioritized. This is necessary to ensure Tribal Warrior is ethically and meaningfully involved as the lead partner during the entire research, co-design, and codevelopment process. Additional time may also be necessary to ensure Tribal Warrior sufficiently understands VR application co-design and codevelopment processes. It is necessary to acknowledge that the VR application content, concepts, and research findings will not represent the broader Aboriginal community or differing groups within the local community context of inner-city Sydney, New South Wales, Australia. Many cultural and community groups exist within inner-city Sydney, New South Wales, Australia, and it is not the intention to be representative of all these groups. Findings will report on cultural relevance, acceptability, and feasibility of using VR to provide SEWB and CDH support. This will enable our research partnership to engage in future research, from formative evidence indicating whether and how participants believe the VR application could be used to provide SEWB and CDH support.

### Conclusions

Little evidence exists on the use of VR to support Aboriginal wellbeing. This study aims to explore, identify, and understand how VR could be used to provide SEWB and CDH support for Aboriginal peoples. Cultural relevance, acceptability, and feasibility evidence will be gained through this study. Indigenist and PAR outcomes will help to better understand the potential of co-designing and codeveloping culturally centered wellbeing support for VR. This study protocol has outlined a 3-phase mixed-methods indigenist and PAR approach that will guide our research.

## Abbreviations

ACCO: Aboriginal Community Controlled Organization
CBT: cognitive behavioral therapy
CDH: cultural determinants of health
DHMS: digital health mental service
eHRCATSIH: eHealth Research Collaboration for Aboriginal and Torres Strait Islander Health
PAR: participatory action research
REDCap: Research Electronic Data Capture
SEWB: social and emotional wellbeing
USYD: University of Sydney
VR: virtual reality
